# The Effect of Treatment With Hyaluronic Acid Dermal Fillers on Skin Changes and Quality of Life in Oncology Patients

**DOI:** 10.1111/jocd.70422

**Published:** 2025-09-04

**Authors:** Eden Bar, Eliya Shachar, Ofir Artzi, Lyri Adar, Omer Shani, Adi Diner, Lee Pantanowitz Mabjeesh, Ido Wolf, Tamar Safra

**Affiliations:** ^1^ Dermatology Department Tel Aviv Sourasky Medical Center Tel Aviv Israel; ^2^ Gray Faculty of Medical & Health Sciences Tel Aviv University Tel Aviv Israel; ^3^ Division of Oncology Tel Aviv Sourasky Medical Center Tel Aviv Israel

**Keywords:** aesthetic treatment, cancer treatment, dermal fillers, quality of life

## Abstract

**Background:**

Patients treated with cancer therapies often experience changes in physical appearance, body image perception, and self‐esteem, which influence their quality of life (QoL).

**Aims:**

To assess the effect of treatment with hyaluronic acid (HA) dermal fillers on QoL, safety, and perceived skin changes of oncology patients.

**Methods:**

A single‐center prospective study conducted between 2021 and 2023 among female oncology patients aged 30–70 years receiving active cancer treatment. All participants completed a questionnaire describing their demographics, disease history, and experience with aesthetic procedures, their intention to undergo aesthetic treatments, and a QoL questionnaire. A subset of participants received treatment with HA fillers. The effect of filler treatment on facial skin features, efficacy, and safety was evaluated by independent dermatologists and by the participants 8–12 weeks after the intervention.

**Results:**

In total, 127 women (median age 57.1 years) with breast (58.3%), gynecological (33.9%), and other cancer types (7.9%) entered the study. Most participants (84.0%) reported no previous treatment with dermal fillers. Thirty‐one participants received a single treatment with HA dermal fillers concurrently with cancer treatment and an optional touch‐up 4 weeks later. At follow‐up, statistically significant improvements in overall aesthetic facial appearance and specific skin quality parameters were observed. Mean QoL significantly improved, with notable improvements in self‐esteem, self‐perception, and overall participant feeling. Adverse events were minimal. Thirteen participants (41.9%) experienced mild dermal events. One participant developed a grade 1 delayed inflammatory reaction 6 months postinjection.

**Conclusions:**

HA fillers have the potential to improve QoL and self‐perception among oncology patients undergoing active cancer therapy.

## 1 Introduction

Body image is multidimensional, encompassing a visual mental representation of one's own external body, along with the internal perceptual, cognitive, and emotional responses associated with one's identity [1, 2]. Cancer treatment involves aggressive interventions such as surgery, radiotherapy and systemic treatments, such as chemotherapy, hormonal, targeted or immunotherapy. These various modalities of care incur skin toxicity including changes in texture, premature aging, volume loss, striae distensae surgical scars, persistent alopecia, radiation tattoos, telangiectasias, hirsutism, and lymphoedema [3, 4]. The burden of treatment has profound effects on patients' physical appearance, often inducing distress and a reduction in self‐reported quality of life (QoL) secondary to changes in body image and self‐esteem [5, 6, 7, 8], which may persist beyond the completion of cancer treatment [9, 10].

Aesthetic treatments enhance body image and positively impact overall QoL. Such treatments have been traditionally intended for healthy individuals aiming to enhance their appearance and mitigate the effects of aging. While these procedures are known to be relatively safe for healthy individuals, their safety among oncology patients treated with active antineoplastic therapies poses unique challenges and uncertainties. Patients undergoing cancer treatments may have compromised immune function and heightened susceptibility to infections [11, 12], raising concerns about potential risks associated with aesthetic interventions. Furthermore, the effects of cancer therapies on tissue healing [13] and vascular function [14] may complicate the safety profile of filler injections in this population. However, recent studies have found no unusual adverse effects associated with filler injections in cancer patients [15].

There is limited data on the effect of dermal fillers administered concomitantly with active cancer treatment. We aimed to assess the effect of treatment with hyaluronic acid (HA) dermal fillers on the QoL, safety, and perceived skin changes of oncology patients undergoing active cancer therapy.

## 2 Methods

### 2.1 Study Design, Setting and Participants

This prospective study was conducted at the Women's Cancer Center and Division of Dermatology at a tertiary medical center between 2021 and 2023 among female oncology patients receiving active cancer treatment at the oncology outpatient center. The study protocol received approval from the institutional ethics committee. Informed consent was obtained from all participants.

Women aged 30–70 years with cancer, Fitzpatrick skin type 2–4 [16] and subjective volume loss or skin deterioration in the face or hands were included in the study. Exclusion criteria included active systemic or local infection, history of extended neutropenia or fever while on therapy, permanent dermal fillers, a known allergy to dermal fillers, a history of vascular, bleeding, collagen, or elastin disorders, a tendency for keloid or hypertrophic scarring, or delayed wound healing. Additional exclusion criteria were recent oral retinoid use, pregnancy or planned pregnancy, and breastfeeding.

All participants were asked to complete a questionnaire addressing demographics, disease history, QoL, perceived skin changes following cancer diagnosis and treatment, previous experience and impression of aesthetic treatments, specifically filler injections. Among the participants who answered the questionnaire, 31 patients were randomly selected to undergo facial HA filler injection to restore volume loss and improve facial wrinkles and lines.

### 2.2 Study Questionnaire

The first part of the questionnaire included information on participants' demographics, disease history, and their experience with aesthetic procedures, specifically HA‐based dermal fillers, and their intention to undergo aesthetic treatments. The participants were also asked to rate statements describing perceptions about filler injections in general and in the context of oncological disease on a Likert scale ranging from 1 (strongly disagree) to 5 (strongly agree), with 0 representing the lack of relevance of the question to the respondent.

In the second part of the questionnaire, the participants were asked to describe changes in their skin (volume, wrinkles, pigmentation, dryness, skin lesion, roughness, glow, and thickness) that had occurred following cancer diagnosis and anticancer treatment and their impact on their QoL. The participants rated each of the skin quality parameters and emotional appearance parameters (tired, angry, and worried) on a 7‐point scale ranging from −3 (marked negative change) to +3 (marked positive change), with 0 indicating no change. This standardized format enabled quantitative evaluation of perceived changes following treatment.

QoL was assessed using the modified Dermatology Life Quality Index (DLQI) [17, 18, 19] which consists of 10 items concerning participants' perception of the impact of skin diseases on different aspects of their health‐related QoL over the past week. Total DLQI ranges from 0 to 30 (0–1: no effect at all on patient's life; 2–5: small effect on patient's life, 6–10: moderate effect on patient's life, 11–20: very large effect on patient's life, 21–30: extremely large effect on patient's life). The questionnaire was completed by all participants at baseline. The 31 participants who underwent treatment with HA fillers also completed the second part of the questionnaire at the follow‐up visit.

### 2.3 Treatment With HA Fillers

Thirty‐one participants who met the eligibility criteria and completed the initial questionnaire were randomly selected to undergo a facial aesthetic treatment with a dermal filler using a computer‐generated random number sequence performed by an independent statistician. Due to ethical considerations, no sham procedure was performed for the comparator group, and as such, the study lacked a blinded control arm.

Each participant received a single treatment with one of the following dermal fillers: Stylage XXL, Stylage XL, Stylage L, or Stylage M (Laboratoires Vivacy, Paris, France). The fillers were injected into one or more of the following facial areas: the midface, nasolabial folds, marionette lines, and oral commissures. The maximal volume of dermal filler per participant was 5 mL. The dermal filler, treatment areas, and injection volume were based on the professional assessment of the treating dermatologist. The dermatologist could perform touch‐up to correct facial asymmetry with a maximum volume of 1 mL of filler in cases where facial asymmetry was observed 4 weeks after treatment. A follow‐up visit was conducted 8–12 weeks after the initial treatment with the HA filler.

To ensure patient safety, all participants in the treatment arm underwent a clinical screening prior to HA filler injection, which included a review of recent complete blood count results. Participants with neutropenia, thrombocytopenia, ongoing infection, or poor general condition were excluded from treatment. In addition, oncologists overseeing each participant's cancer treatment were informed and involved in approving the timing and safety of the aesthetic intervention.

### 2.4 Evaluation of Aesthetic Improvement Following Treatment With HA Fillers

The participants were photographed in the same position and under the same lighting conditions using the VISIA Imaging System (Canfield Scientific Inc. Fairfield, NJ, USA) and a Single‐Lens Reflex digital camera (EOS 600D, Canon Tokyo, Japan) before the HA filler injection (baseline) and at the follow‐up visit (8–12 weeks after the initial treatment).

All photographs were de‐identified and randomly ordered prior to evaluation by three independent dermatologists who were blinded to the timepoint of the photo (baseline or follow‐up) and had no access to clinical information, thereby ensuring unbiased assessments.

The three independent dermatologists rated the change in severity of the nasolabial folds, marionette lines, oral commissures, and midface volume, using the Modified Fitzpatrick Wrinkle Scale (MFWS) [20], Marionette Lines Grading Scale [21], Oral Commissures Severity Scale [22], and Medici's Midface Volume Deficit Scale (MMVDS) [23], respectively. These scales range from 0 (none) to 4 (most severe). The participants' overall change in appearance was evaluated by the dermatologists using the Global Aesthetic Improvement Scale (GAIS) [24], on the following scale: −1 (worse), 0 (no change), 1 (minor improvement), 2 (moderate improvement), 3 (significant improvement). The dermatologists also rated each photo for their perceived impressions of patient illness, sadness, distraction, and coping abilities using a scale ranging from 1 (strongly disagree) to 8 (strongly agree). Adverse events following treatment were recorded and graded.

### 2.5 Statistical Analysis

The data were analyzed using R version 4.4.1 (R Development Core Team. Vienna, Austria). Numeric variables were summarized with median and range. Categoric variables were summarized with frequencies and percentages. Changes from baseline were tested for significance using the one‐sample *t*‐test. All tests applied were two‐tailed, and a *p*‐value of 5% or less was considered statistically significant.

## 3 Results

### 3.1 Study Population

A total of 127 women with breast (58.3%), gynecological (33.9%), and other cancer types (7.9%) were included in the study. The median age of the study participants was 57.1 years (range 29.4–88.4). The most common malignancy among the participants was breast cancer (58.3% of participants) followed by ovarian cancer (22.1%). Most participants (60.0%) entered the study with advanced disease, stage IV. All study participants received systemic therapy for malignant disease, including chemotherapy, targeted therapy, immunotherapy, and hormonal therapy ‐ alone or in combination. The median duration of anticancer treatment was 10.5 months (range 1.4–54.5), with a median number of 2 treatment lines (range 1–8) (Table 1).

**TABLE 1:** Clinical and demographic characteristics of the study population.

Parameter	Treated with HA fillers *N* = 31	Untreated *N* = 96	All *N* = 127
Age, years, median (min, max)	51.5 (29.4–71.7)	58.8 (30.2, 88.4)	57.1 (29.4, 88.4)
Primary cancer, *n*/*N* (%)			
Breast	24/31 (77.4%)	50/96 (52.1%)	74/127 (58.3%)
Ovary	4/31 (12.9%)	24/96 (25%)	28/127 (22.1%)
Uterus	2/31 (6.5%)	7/96 (7.3%)	9/127 (7.1%)
Cervix	0/31 (0%)	6/96 (6.3%)	6/127 (4.7%)
Other^a^	1/31 (3.2%)	9/96 (9.4%)	10/127 (7.9%)
Stage at diagnosis, *n*/*N* (%)			
Stages I–III	12/30 (40.0%)	77/91 (42.8%)	51/121 (42.14%)
Stage IV	18/30 (60.0%)	52/91 (57.14%)	70/121 (57.85%)
Type of treatment, *n*/*N* (%)			
Chemotherapy with/without targeted therapy/immunotherapy/hormonal treatment	19/31 (61.3%)	53/79 (67.1%)	72/110 (65.5%)
Hormonal treatment	6/31 (19.4%)	8/79 (10.1%)	14/110 (12.7%)
Targeted therapy	3/31 (9.7%)	11/79 (13.9%)	14/110 (12.7%)
Immunotherapy with/without targeted therapy	2/31 (6.5%)	4/79 (5.1%)	6/110 (5.5%)
Targeted, hormonal therapy	1/31 (3.2%)	3/79 (3.8%)	4/110 (3.6%)

Abbreviation: HA, hyaluronic acid.

^a^ Dendroglioma, cell carcinoma, adenocarcinoma, cutaneous marginal zone lymphoma, transitional cell carcinoma, pleomorphic adenocarcinoma, and colon cancer.

The baseline and clinical characteristics of the 31 participants who were randomly selected to receive HA fillers were similar to those of the general study population (Table 1).

### 3.2 Participants' Aesthetic Experience and Conceptions

Most participants (106/127, 83.5%) reported no previous treatment with dermal fillers. The most common reason for avoiding this aesthetic treatment was their malignant disease (57/106, 53.8%). A third of these participants (34/106, 32.1%) reported that their decision originated from concerns shared by other patients, or advice from their treating oncologist (43/106, 40.6%) suggesting they refrain from aesthetic treatments. Approximately half of the participants (57/106, 53.8%) indicated they may consider filler injections in the future after achieving remission.

Twenty‐one participants (16.5%) were previously treated with dermal fillers; most (14/21, 66.7%) reported that they had received 2–3 prior treatments with HA fillers. Fifteen patients (71.4%) received filler injections before their cancer diagnosis. Six patients (28.6%) were treated with dermal fillers concurrently with an active disease—three of them concomitantly with the administration of chemotherapy. Among the 21 participants who had received filler injections prior to this study, 13 (61.9%) did not inform the treating aesthetic physician about their malignancy, and 11 (52.4%) did not inform their treating oncologist about the aesthetic intervention. Additionally, 18 (85.7%) reported that their aesthetic physician did not consult their oncologist before the procedure. Most participants (15/21, 71.42%) inquired about the expertise of the injecting physician and the nature of the filler injected (17/21, 80.9%). Financial considerations when choosing the injecting physician were deemed less significant by 76.19% (16/21) of participants.

### 3.3 Aesthetic Outcomes Following Treatment With HA Fillers

The median age of the 31 participants who were randomly chosen to receive treatment with HA fillers was 51.5 years (range 29.4–71.7). At the time of the aesthetic treatment, the participants' median anticancer therapy line was 2 (range 1–8) and the median time on the current therapy was 10.5 weeks (median 1.4–54.5). The mean “before” versus “after” scores of the facial areas treated, as evaluated by three independent dermatologists, are shown in Table 2. Statistically significant improvements from baseline to follow‐up were observed in nasolabial folds, marionette lines, oral commissures, and midface, as well as in total appearance. Furthermore, at follow‐up, the participants were perceived by the dermatologists as being less sick, sad, and distracted, and better able to cope. At baseline, prior to treatment with the dermal fillers, negative changes in facial volume were reported by 45.2% of the 31 participants treated with HA fillers, more wrinkles (64.5%), increased pigmentation (48.4%), reduced skin hydration (58.1%), decreased skin glow (51.6%) and decreased skin thickness (48.4%). At follow‐up, 8–12 weeks after the initial HA filler injections, notable positive changes were reported by the participants, including volume repletion (21.4% of participants), a reduction in wrinkles (29.6%), improved skin hydration (23.1%), enhanced skin glow (23.1%), and increased skin thickness (23.1%). Changes in perceived appearance were also reported: 53.6% of participants reported looking less tired, 38.5%—less angry, and 34.6%—less worried compared to 74.2%, 48.4%, and 32.3% of participants who perceived their appearance as tired, angry, and worried, respectively, prior to treatment with the HA fillers.

**TABLE 2:** Change in aesthetic appearance of facial regions injected with HA fillers, as assessed by three independent dermatologists.

Facial region	Scale	Baseline mean ± SD	Follow‐up mean ± SD	Change mean ± SD	*p*
Nasolabial folds	MFWS 0 (none) to 4 (severe)	2.74 ± 1.02	1.6 ± 0.73	−1.14 ± 0.47	< 0.001
Marionette lines	Marionette Lines Grading Scale 0 (none) to 4 (severe)	2.45 ± 0.99	1.23 ± 0.6	−1.23 ± 0.64	< 0.001
Oral commissures	OCSS 0 (none) to 4 (severe)	2.21 ± 1.16	1. ± 0.67	−1.21 ± 0.7	< 0.001
Midface	MMVDS 0 (none) to 4 (severe)	2.45 ± 0.85	1.44 ± 0.7	−1.01 ± 0.56	< 0.001
Overall facial appearance	GAIS −1 (worse) to 3 (significant improvement)	NA^a^	2.21 ± 0.67	2.21 ± 0.67	< 0.001
Perceived impression					
Sick	1 (strongly disagree) to 8 (strongly agree)	4.67 ± 1.15	2.93 ± 0.7	−1.74 ± 1.07	< 0.001
Sad	1 (strongly disagree) to 8 (strongly agree)	4.27 ± 0.93	2.82 ± 0.7	−1.45 ± 0.92	< 0.001
Distracted/anxious	1 (strongly disagree) to 8 (strongly agree)	3.88 ± 1.26	2.38 ± 0.65	−1.5 ± 1.05	< 0.001
Unable to cope	1 (strongly disagree) to 8 (strongly agree)	4.39 ± 1.11	2.35 ± 0.64	−2.05 ± 1.35	< 0.001

Abbreviations: GAIS, Global Aesthetic Improvement Scale; MFWS, Modified Fitzpatrick Wrinkle Scale; MVDSS, Medici Midface Volume Deficit Scale; NA, not applicable; OCSS, Oral Commissures Severity Scale; SD, standard deviation.

^a^ GAIS only evaluates change from baseline; therefore, there is no baseline score.

The mean total score of the DLQI for participants who received HA filler treatment statistically significantly decreased from 12.4 ± 5.8 at baseline to 5.1 ± 4.1 at follow‐up (*p* = 0.006), indicating a significant improvement in their QoL posttreatment.

Most participants (80.8%) expressed high satisfaction following treatment with HA fillers, with 75% affirming a desire for future HA filler treatments.

### 3.4 Adverse Events of HA Filler Treatment

Thirteen participants (41.9%) experienced minor transient cutaneous side effects (grade 1) after the HA injection, which included post‐procedural pain, swelling, bruises, and redness (Table 3). One participant developed a grade 1 delayed inflammatory reaction 6 months postinjection, which occurred shortly after a dental procedure. The treatment of the delayed inflammatory reaction involved a 2‐month course of minocycline. No severe or serious adverse events were reported in any of the participants.

**TABLE 3:** Adverse events.

Adverse event	Treated population *N* = 31 *n* (%)
**Participants with at least one adverse event**	13 (41.9%)^**a**^
Swelling	6 (19.35%)
Tenderness	4 (12.9%)
Bruising	4 (12.9%)
Redness	3 (9.67%)
Skin induration/lump or irregularity	2 (6.5%)
Prolonged erythema	1 (3.2%)
Skin hypopigmentation	1 (3.2%)
Delayed inflammatory reaction (6 months posttreatment)	1 (3.2%)

^a^ All adverse events were grade 1.

## 4 Discussion

Many studies have highlighted the negative impact of cancer and its treatments on patients' self‐esteem and QoL [9, 25, 26, 27]. Body image concerns, emotional distress, coping challenges and lower QoL are commonly reported among cancer patients [28, 29, 30]. While aesthetic treatments, including fillers, may play a significant role in addressing both the physical and psychological aspects of patient care, many physicians are unaware of the potential benefits of treatment with HA fillers for individuals with cancer, and often refrain from suggesting them due to concerns about the complexity of managing cancer patients' cases and potential adverse effects both from the treatment itself and the concomitant cancer treatment.

This study demonstrated that treatment with HA fillers improves the overall facial appearance of women with cancer, including volume, wrinkles, skin hydration, skin glow, and skin thickness, as well as their perceived appearance and QoL with minimal adverse effects. Satisfaction rates were high, with notable improvements in self‐esteem, self‐perception, and overall participant feelings.

Our study corroborates the findings of other studies, showing that aesthetic procedures can significantly improve the physical and psychological well‐being, as well as the QoL of individuals with chronic illnesses, including cancer. Dayan et al. [31] demonstrated that aesthetic treatments have broader psychosocial benefits that extend beyond physical appearance to enhance social interactions and perceptions. Evidence also suggests that nonpermanent fillers are effective and safe for treating facial atrophy associated with chronic illnesses, such as connective tissue disease, acquired immunodeficiency syndrome (AIDS) [32], and autoimmune inflammatory rheumatic diseases [33].

The application of HA fillers in oncology patients is an emerging field. To date, most documented cases have involved treatments administered to cancer survivors rather than to patients actively undergoing cancer therapy. Shamban [34] reported significant improvements in wrinkle severity with minimal adverse events among post‐chemotherapy patients who received facial rejuvenation treatments with HA. Egidi et al. [35] reported a case of successful HA filler use in a cancer survivor with postradiation orbital defects, demonstrating improvements not only in aesthetic outcomes but also in functionality (eyelid closure, hydration, and visual acuity). Similarly, Schnorr et al. [36] described the use of botulinum toxin type A, HA, and calcium hydroxylapatite in 5 oncologic patients to achieve prosthetic rehabilitation refinement prior to manufacturing oculofacial prostheses. These reports reinforce the potential benefits of dermal fillers for restoring self‐image and enhancing the QoL and emotional and psychological well‐being of oncologic patients [15], consistent with our research outcomes. Our study is unique in showing that dermal fillers can be used while patients are undergoing active cancer treatment. Sung et al. also described a single case of a patient successfully treated with HA filler injection while undergoing imatinib mesylate treatment for chronic myeloid leukemia [37]. These studies consistently reinforce the view that aesthetic rehabilitation should be integrated into oncology patient and survivorship care with strict attention to safety, immunological status, and multidisciplinary coordination.

The adverse events observed in this study are expected of this type of filler. Although within an acceptable range, the 42% incidence of minor adverse events is somewhat higher than typically reported in healthy populations (about 20%–40%) [38, 39]. This slightly higher rate of minor adverse events may reflect the altered dermal milieu and healing capacity in actively treated oncology patients. A single delayed inflammatory response occurred in one participant 6 months after the intervention following dental treatment. This is a well‐documented potential trigger for delayed filler‐related responses and suggests possible immune cross‐reactivity. Based on this observation, we have implemented stricter posttreatment monitoring, closer clinical follow‐up, and pre‐procedural counseling to minimize such risks and improve patient safety.

Our work has several limitations, including a small sample size and reliance on participant‐reported information. We fully acknowledge the limitations imposed by the absence of a parallel control group undergoing a placebo or sham procedure. However, our study was exploratory in nature, aimed at assessing real‐world safety, QoL outcomes, and feasibility of aesthetic treatments in actively treated oncology patients—a population traditionally excluded from such interventions. The ethical and logistical challenges of performing a sham injection procedure in vulnerable cancer patients made a placebo‐controlled design impractical at this stage.

Further research using a rigorous study design, such as a randomized controlled trial, and a larger and more diverse patient population, are warranted to validate these findings and guide safe, evidence‐based integration of aesthetic care into oncologic practice.

## 5 Conclusion

This study highlights the potential value of HA dermal fillers treatments in improving the QoL and self‐perception among oncology patients undergoing active cancer therapy. However, given the study's limitations, including the lack of a control group, the small sample size, and reliance on patient‐reported outcomes, these results should be interpreted as preliminary. We recommend future controlled, multicenter studies with larger and more diverse patient populations to validate these findings and guide safe, evidence‐based integration of aesthetic care into oncologic practice.

## Author Contributions

O.A. and T.S. conceptualized and designed the study, collected and analyzed the data, wrote and revised the manuscript. E.B., E.S., L.A., O.S., A.D., and L.P.M. collected the data. I.W. critically revised the manuscript. All authors approved the final version of the manuscript.

## Ethics Statement

The study protocol received approval from Tel Aviv Sourasky's Ethics Committee (0313‐21‐TLV). Informed consent was obtained from all participants.

## Conflicts of Interest

The authors declare no conflicts of interest.

## Data Availability

The data that support the findings of this study are available from the corresponding author upon reasonable request.
